# Unraveling early redox responses and secondary metabolite accumulation in garlic under drought and high temperature

**DOI:** 10.3389/fpls.2026.1860406

**Published:** 2026-07-31

**Authors:** Tvrtko Karlo Kovačević, Nikola Major, Marina Krpan, Dean Ban, Anja Batel, Mario Franić, Smiljana Goreta Ban

**Affiliations:** 1 Department of Agriculture and Nutrition, Institute of Agriculture and Tourism, Poreč, Croatia; 2 Centre of Excellence for Biodiversity and Molecular Plant Breeding, Zagreb, Croatia; 3 Department of Nutrition, Quality and Food Safety, Faculty of Food Technology and Biotechnology, University of Zagreb, Zagreb, Croatia

**Keywords:** *Allium sativum*, Flavonoids, organosulfur compounds, polyphenolics, redox state

## Abstract

Garlic (*Allium sativum* L.) is valued for its nutritional, economic, and bioactive properties, yet its secondary metabolic responses to concurrent water-availability and temperature conditions remain poorly understood. This study investigated the temporal metabolic responses of garlic plants to drought and high temperature, applied individually and in combination, over an 8-day period, using univariate and multivariate approaches. Drought emerged as the dominant factor influencing plant redox status, leading to a pronounced shift in glutathione redox status by day 8, characterized by increased content of oxidized glutathione (GSSG), proportion of oxidized glutathione within the total glutathione pool (%GSSG), and a decreased reduced-to-oxidized glutathione ratio (GSH/GSSG). These changes indicate enhanced oxidative pressure and activation of redox-associated signaling pathways. Methyl jasmonate (MeJA) accumulation, particularly under co-occurring drought and high temperature, was temporally associated with increased contents of several flavonoid glycosides, suggesting a link between jasmonate-related signaling and phenylpropanoid-derived antioxidant responses. Conversely, organosulfur metabolites displayed variable responses, reflecting complex regulation and potential sensitivity to temperature. Multivariate analysis further revealed a time- and treatment-dependent shift in metabolic response, with water availability becoming the primary source of data variation towards the end of the experiment. These findings highlight the importance of stress interactions and temporal dynamics in shaping plant early metabolic responses, and point to glutathione redox parameters and MeJA as candidate metabolite-level indicators warranting further validation for stress monitoring.

## 1 Introduction

Garlic (*Allium sativum* L.) is one of the most widely cultivated vegetables worldwide, valued for both its economic and nutritional significance (Badran, 2015). Beyond its role as a staple ingredient in culinary traditions around the world, garlic is also renowned for its rich content of bioactive compounds, which contribute to its health-promoting properties, including antioxidant, antimicrobial, and cardioprotective effects (El-Saadony et al., 2024). Its dual role as a valuable specialty crop and a functional food highlights its importance in both agricultural economies and human nutrition (El-Saadony et al., 2024).

In the Mediterranean region, the anticipated rise in the frequency and intensity of droughts and high temperatures due to global climate change poses a significant threat to agricultural productivity, crop quality, and long-term food security (Giorgi and Lionello, 2008). Importantly, such environmental conditions rarely occur in isolation; in natural field conditions, plants are frequently exposed to multiple concurrent environmental conditions (Xu et al., 2023). For plants, unfavorable environmental conditions, such as drought, flooding, heat, increased salinity, etc., are in literature described as abiotic stressors (Zhang et al., 2023). Drought coinciding with high temperature, leads to complex physiological and biochemical responses that cannot be predicted by studying each stressor independently (Xu et al., 2023). As a result, understanding plant responses to multiple environmental conditions, is essential for accurately predicting the impact of climate change on crop resilience and for developing effective mitigation strategies (Xu et al., 2023). This is particularly relevant for regions such as the Mediterranean, where garlic is traditionally cultivated and widely consumed, and where drought and high temperature events increasingly threaten crop productivity and quality (Shanker et al., 2014).

Secondary metabolites such as polyphenolic compounds constitute an extensive and diverse class of plant compounds that are crucial for plant survival under varying environmental conditions (Anjali et al., 2023; Dias et al., 2021). Phenolic acids, including *p*-coumaric acid and ferulic acid are well-recognized components of the phenylpropanoid pathway, acting as antioxidants that mitigate reactive oxygen species (ROS) generated during drought and high temperature, which in turn trigger cellular signaling pathways governed by phytohormones (Ahmad et al., 2016; Ramaroson et al., 2022). Among these, jasmonic acid and its volatile derivative methyl-jasmonate are rapidly activated in response to unfavorable environmental stimuli, functioning as key mediators amplifying cellular signals and inducing the biosynthesis of polyphenolic compounds, thereby enhancing the plant’s adaptive capacity (Pradhan et al., 2024; Qaderi et al., 2023; Wasternack and Strnad, 2019). The interplay between environmental conditions results in complex metabolite dynamics, reflecting phased activation of biochemical pathways, such as the phenylpropanoid and flavonoid biosynthesis routes (Ninkuu et al., 2025; Zhang et al., 2023). In addition to polyphenolic, *Allium* species synthesize organosulfur compounds such as alliin, deoxyalliin, and allicin, which are pivotal for both basal metabolic functions and adaptive biochemical mechanism to unfavorable environmental conditions (Amagase, 2006; Borlinghaus et al., 2014).

Given garlic’s economic and nutritional value, elucidating how polyphenolic and organosulfur pathways are reprogrammed under concurrent environmental conditions is critical for predicting phytochemical outcomes under field scenarios. Accordingly, this study employs a factorial design to explore the effects of individual and concurrent drought and high temperature conditions on garlic’s metabolic dynamics of phytohormones, redox related signaling components, polyphenolic and organosulfur compounds. We hypothesized that combined drought and high temperature would induce a metabolic response in garlic leaves that differs from the response to each individual stress. Therefore, the objective of this study was to characterize short-term temporal changes in these metabolite classes using targeted LC-MS/MS profiling and to identify treatment-associated metabolite patterns under drought and/or high temperature conditions. Moreover, shifts in these metabolic markers could offer strong potential in field application as biochemical indicators for stress monitoring, and support for the development of climate-resilient production systems.

## 2 Materials and methods

### 2.1 Plant material and experimental design

Outermost cloves of the selected garlic (*Allium sativum* L.) cultivar Istarski crveni were obtained from the gene bank of the Institute of Agriculture and Tourism (Poreč, Croatia), where bulbs had been maintained under cool, dry conditions throughout the natural post-harvest dormancy period prior to planting. A total of 600 uniform, large outer cloves were planted on September 25th, 2023, in polystyrene containers filled with a peat–vermiculite substrate (8:5, v/v). Plants were initially cultivated in an unheated greenhouse at the same institute (45°13′20.351″N, 13°36′6.397″E), where standard irrigation practices were applied and emergence was monitored. After 40 days, 300 uniform and healthy plants (75 per treatment) were transferred to controlled-environment growth chambers (GC-401, Nüve, Turkey) and acclimated under a 16 h light/8 h dark photoperiod, 22/18 °C day/night temperatures, and 50% relative humidity, following the conditions described by Bauer et al (Bauer et al., 2022). A two-factorial experimental design was then implemented to assess the combined effects of water availability and temperature. One chamber was adjusted to high temperature conditions (38/24 °C day/night; 16 h light/8 h dark; 50% relative humidity), with gradual transitions at the onset of each photoperiod to simulate natural diurnal changes, while the second chamber maintained ambient conditions. Drought stress was imposed simultaneously by withholding irrigation from half of the plants within each chamber. The selected stress intensities were based on literature data and preliminary optimization trials to ensure physiologically relevant and reproducible conditions. This resulted in four treatment groups (n = 75): water-available and ambient temperature (W+/T−), water-available and high temperature (W+/T+), drought-induced and ambient temperature (W−/T−), and drought-induced and high temperature (W−/T+). Over the 8-day experimental period, substrate water content was monitored gravimetrically to maintain distinct moisture regimes, and 4 plants per treatment were sampled at each time point for bioactive compound analyses. Additionally, 3 plants per sampling point were oven dried at 105 °C in an oven (Memmert UF160, Schwabach, Germany) until constant mass to calculate the obtained metabolite content results per fresh weight basis.

### 2.2 Substrate water content monitoring

Substrate from each treatment (W+/T-, W-/T-, W+/T+, and W-/T+) were taken and water content was measured by calculating the difference between the masses of the substrate before and after drying in a forced hot air circulation oven (Memmert UF160, Schwabach, Germany) held at 105 °C according to ISO11465:1993 until constant weight (**Table 1**).

**Table 1:** Substrate water content measurements of garlic plants exposed to water-available and ambient temperature (W+/T-), water-available and high temperature (W+/T+), drought-induced and ambient temperature (W-/T-), and drought-induced and high temperature conditions (W-/T+) across 8-day experimental period (mean ± SE, *n* = 4).

Substrate water content (%)					
days	W+/T+	W+/T-	W-/T+	W-/T-	*p*-value
1	74.2 ± 0.6 A^1^:a^2^	69.7 ± 0.7 B:a	63.0 ± 1.6 C:a	68.3 ± 1.3 B:a	***
2	67.7 ± 0.9 A:b	50.7 ± 2.9 C:d	55.6 ± 1.5 C:a	63.9 ± 0.6 B:a	***
3	51.2 ± 4.1 -:d	54.9 ± 3.9 -:d	41.0 ± 2.7 -:bc	44.2 ± 0.7 -:b	ns
4	57.2 ± 0.3 B:cd	60.1 ± 0.6 A:cd	44.7 ± 3.2 C:b	45.0 ± 1.6 C:b	***
5	59.5 ± 0.8 A:c	64.5 ± 2.2 A:bc	41.2 ± 5.8 B:bc	25.9 ± 2.1 B:c	***
6	66.3 ± 0.5 A:b	65.7 ± 2.2 A:ab	34.4 ± 0.9 B:cd	25.5 ± 0.9 C:d	***
7	67.4 ± 1.1 A:b	69.2 ± 1.0 A:ab	30.3 ± 2.7 B:de	21.9 ± 0.9 C:e	***
8	65.1 ± 1.4 B:b	70.3 ± 0.4 A:a	21.6 ± 3.8 D:e	36.7 ± 1.5 C:d	***
*p*-value	***	**	***	***	

^1^Capital letters indicate significant differences between treatments within days in Tukey’s *post-hoc* test; ns—not significant; *p < 0.05; **p < 0.01; ***p < 0.001,.

^2^Small letters indicate significant differences between days within treatment groups in Tukey’s *post-hoc* test; ns—not significant; *p < 0.05; **p < 0.01; ***p < 0.001.

### 2.3 Extraction of bioactive compounds

The extraction of the metabolites was performed according to Kovačević et al (Kovačević et al., 2023). Briefly, fully expanded young and healthy garlic leaves (4 biological replicates per treatment group) were immediately flash-frozen in liquid nitrogen and lyophilized for 48 hours using a freeze dryer (Labogene ScanVac CoolSafe, Allerød, Denmark). From each lyophilized sample, 100 mg of leaf tissue was accurately weighed and subjected to sequential extraction in five steps using 1 mL of 80:20 methanol:water (v/v) at 30 °C. Homogenization was carried out with a bead mill homogenizer (Omni International, Kennesaw, GA, USA) operating at 6 m/s for 2 minutes. Following each extraction step, samples were centrifuged at 16,000 × g for 10 minutes (Domel Centric 350, Železniki, Slovenia), and the resulting supernatant was transferred to a clean tube for further analysis.

### 2.4 Metabolite identification and quantification

Following the extraction of samples, an aliquot was separated and dried in a lyophilizer (Labogene ScanVac CoolSafe, Allerød, Denmark) and then reconstituted by adding H_2_O and 0.1% formic acid. The phytochemical profile was analyzed on an LC-MS/MS, which consisted of an autosampler (Shimadzu Nexera SIL-40CX3, Kyoto Japan), two solvent delivery units (Shimadzu Nexera LC-40DX3, Kyoto, Japan), a thermostatic column compartment (Shimadzu Nexera CTO-40C, Kyoto, Japan), and a triple quadrupole mass spectrometer (Shimadzu LCMS8045, Kyoto, Japan). The separation of polyphenolics was performed according to Major et al (Major et al., 2023), on a C18, 2.1 mm × 150 mm, 2.7 µm core–shell column (Advanced Materials Technology, Wilmington, DE, USA) held at 37 °C by injecting 1 µL of the sample using a linear gradient elution of mobile phase A (water/0.1% acetic acid) and mobile phase B (methanol/0.1% acetic acid) at 0.35 mL/minute, for 0 min to 0.75 min: 98%A; 0.75 min to 15 min: 98%A to 50%A; 15 min to 15.1 min: 50%A to 0%A; 15.1 min to 20 min: 0%A; 20 min to 20.1 min: 0%A to 98%A; and 20.1 min to 25 min: 98%A. The analysis of reduced and oxidized glutathione, organosulfur compounds, and phytohormone profile was performed according to Polić Pasković et al (Polić Pasković et al., 2024), by achieving separation on a Discovery^®^ HS F5–3 column (2.1 mm × 150 mm, 3 µm core–shell, Sigma-Aldrich, St. Louis, MO, USA) at 37 °C. A 1 µL sample was injected, and separation was achieved using a linear gradient of mobile phase A (water with 0.1% formic acid) and mobile phase B (acetonitrile with 0.1% formic acid) at a flow rate of 0.25 mL/min. The gradient program was as follows: 0–2 min: 100% A; 2–5 min: 100% A to 75% A; 5–11 min: 75% A to 65% A; 11–15 min: 65% A to 5% A; 15–20 min: 5% A; 20–20.1 min: 5%A to 100% A; and 20.1–25 min: 100% A. The identification and quantification of targeted compounds were performed by comparing retention times, characteristic precursor/product ion pairs, and peak areas to reference standards. Ratio of reduced glutathione to oxidized glutathione was calculated according to the formula:


$$\frac{\left[GSH\right]}{\left[GSSG\right]}.$$

The proportion of oxidized glutathione within the total glutathione pool was calculated as follows:


$$\%GSSG=\;\frac{2\;x\;\left[GSSG\right]}{\left[GSH\right]+2\;x\left[GSSG\right]}\;x\;100.$$

### 2.5 Statistical analysis

The obtained data were tested for normality of residuals and homogeneity of variances using the Kolmogorov-Smirnov and Levene’s tests, respectively. A two-way analysis of variance (two-way ANOVA) was conducted to examine the effects of main factors, water-availability and temperature regime, along with their interaction on investigated variables. As interaction proved to be significant for each day, a one-way analysis of variance (one-way ANOVA) was conducted to investigate the differences between plants exposed to different conditions within days and between days within group. As homogeneity of variances was satisfied for all variables in the dataset, all variables were analyzed using the same statistical method, noting that ANOVA is generally robust to moderate deviations from normality, especially in balanced designs (n = 4 per group) (Schmider et al., 2010). For variables where significant differences were detected between plants exposed to different conditions within days and between days within group, Tukey’s HSD test was applied as a *post-hoc* analysis. Given the exploratory nature of running separate one-way ANOVAs per metabolite per day, unadjusted *p*-values are reported, and results should be interpreted with caution regarding potential family-wise error rate inflation. Partial least squares discriminant analysis (PLS-DA) was further employed as an exploratory supervised multivariate method to visualize treatment separation and identify the most important metabolites contributing to differentiation of the treatment conditions (W+/T−, W+/T+, W−/T−, W−/T+), and as a complementary support for univariate analyses. The PLS-DA models were developed using 1000 iterations, while model performance was evaluated using V-fold cross-validation. All statistical analyses were conducted using XLSTAT (2007), a statistical software for Excel. Additionally, pathway analysis was performed in MetaboAnalyst 6.0, using the identified targeted metabolites to evaluate pathway-level enrichment and topological impact (Pang et al., 2024).

## 3 Results

### 3.1 Univariate analysis

**Figure 1:**
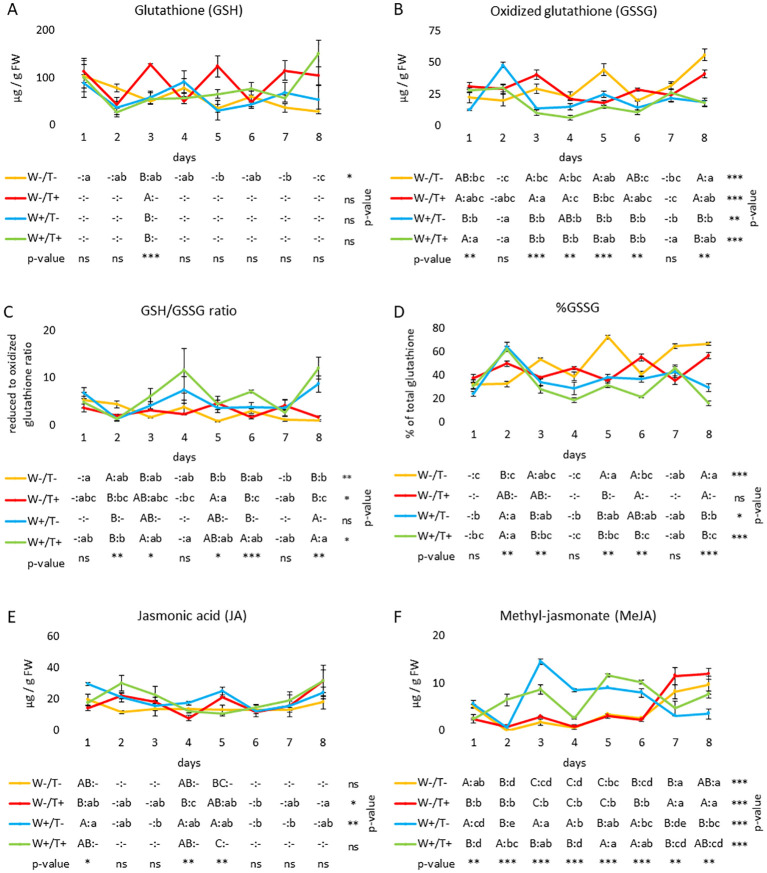
**(A)** GSH, **(B)** GSSG, **(C)** GSH/GSSG ratio, **(D)** %GSSG, **(E)** JA, and **(F)** MeJA content of garlic plants exposed to water-available and ambient temperature (W+/T-), water-available and high temperature (W+/T+), drought-induced and ambient temperature (W-/T-), and drought-induced and high temperature conditions (W-/T+). Capital letters indicate significant differences between treatments within days in Tukey’s *post-hoc* test, while small letters indicate significant differences between days within treatment groups in Tukey’s *post-hoc* test. ns, not significant; * *p* < 0.05; ** *p* < 0.01; *** *p* < 0.001.

The significant differences in oxidized glutathione (GSSG) content observed before day 4 are unlikely to reflect water deficit given that substrate water content in garlic plants exposed to drought (W−/T+ and W−/T−) were comparable to the watered treatments (W+/T− and W+/T+) until day 4 (**Table 1**). In addition, during the same period, no clear effect of high temperature was observed as well. By the end of the 8-day experiment, garlic plants exposed to drought (W−/T+ and W−/T−) had significantly higher GSSG content compared to watered plants (W+/T- and W+/T+). This was also reflected in a significantly higher proportion of GSSG within the total glutathione pool (%GSSG), and in a significantly lower GSH/GSSG ratio on day 8 compared to watered plants (W+/T− and W+/T+). Taken together with the significantly lower reduced glutathione (GSH) and higher GSSG content observed within W−/T− plants on day 8, compared to the previous days, indicates a change in cellular redox state towards more oxidized cellular environment (**Figures 1A–D**). Despite the increase in substrate water content (**Table 1**) from day 4 onward, jasmonic acid (JA) content in drought-exposed plants (W−/T− and W−/T+) was comparable to watered plants at ambient (W+/T-), except for significantly lower JA content on days 1 and 4 in W−/T+ plants and on day 5 in W−/T− plants (**Figure 1E**). Nevertheless, plants exposed to drought (W−/T− and W−/T+) had significantly lower methyl-jasmonate (MeJA) content compared to watered plants at ambient temperature (W+/T−) from day 4 to day 6. On the following days (7 and 8), MeJA content increased significantly, but only in garlic plants exposed to simultaneous drought and high temperature (W-/T+), while it was comparable under individual stresses (W−/T− and W+/T+) relative to W+/T− plants, respectively (**Figure 1F**). In addition, on days 7 and 8, MeJA content within drought-exposed groups (W−/T+ and W−/T−) increased significantly compared to previous time points (except on day 1 in W−/T− plants).

**Figure 2:**
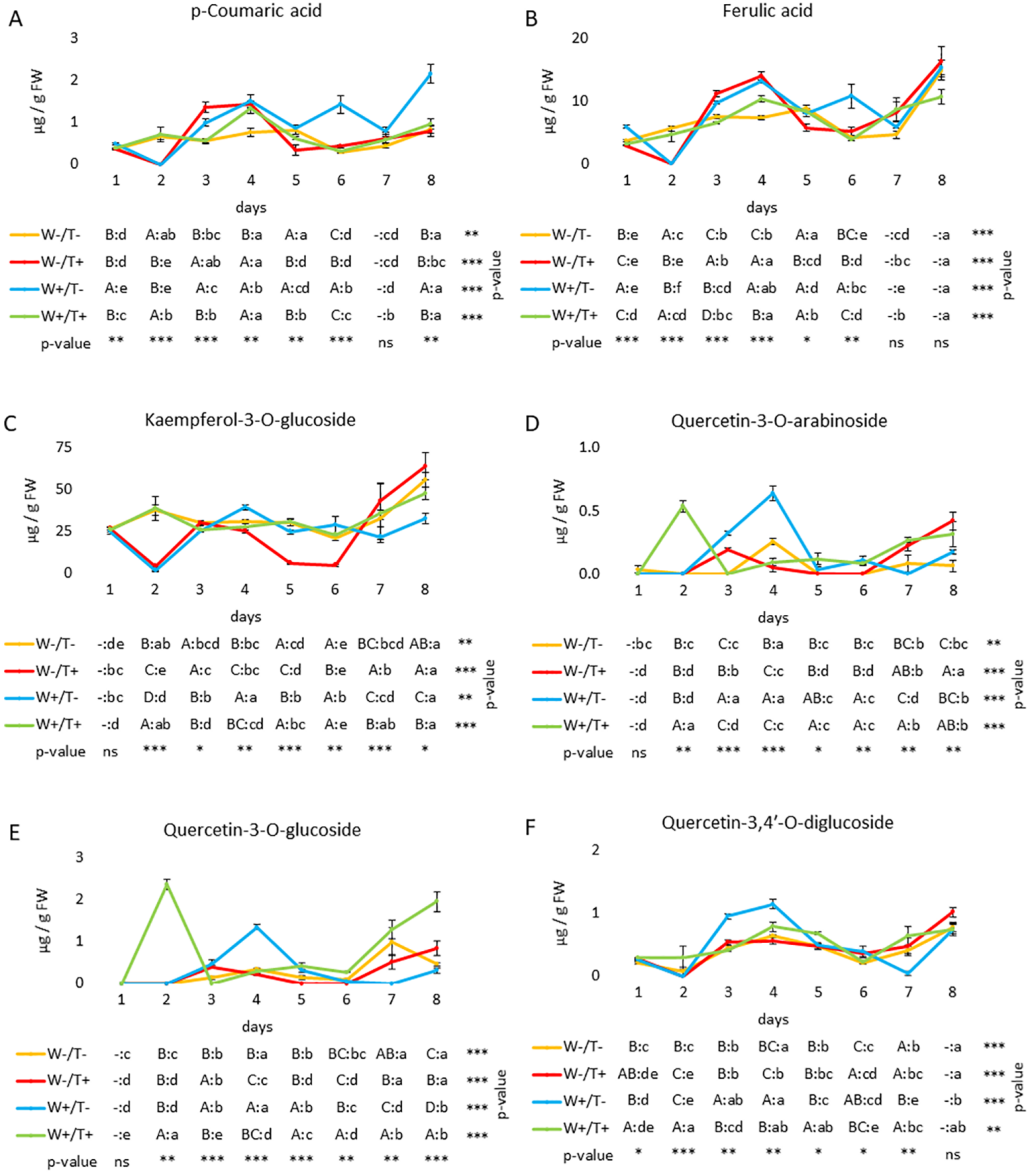
**(A)** p-Coumaric acid, **(B)** Ferulic acid, **(C)** Kaempferol-3-O-glucoside, **(D)** Quercetin-3-O-arabinoside, **(E)** Quercetin-3-O-glucoside, and **(F)** Quercetin-3,4′-O-diglucoside content in leaves of garlic plants exposed to water-available and ambient temperature (W+/T-), water-available and high temperature (W+/T+), drought-induced and ambient temperature (W-/T-), and drought-induced and high temperature conditions (W-/T+). Capital letters indicate significant differences between treatments within days in Tukey’s *post-hoc* test, while small letters indicate significant differences between days within treatment groups in Tukey’s *post-hoc* test. ns, not significant; * *p* < 0.05; ** *p* < 0.01; *** *p* < 0.001.

**Figure 3:**
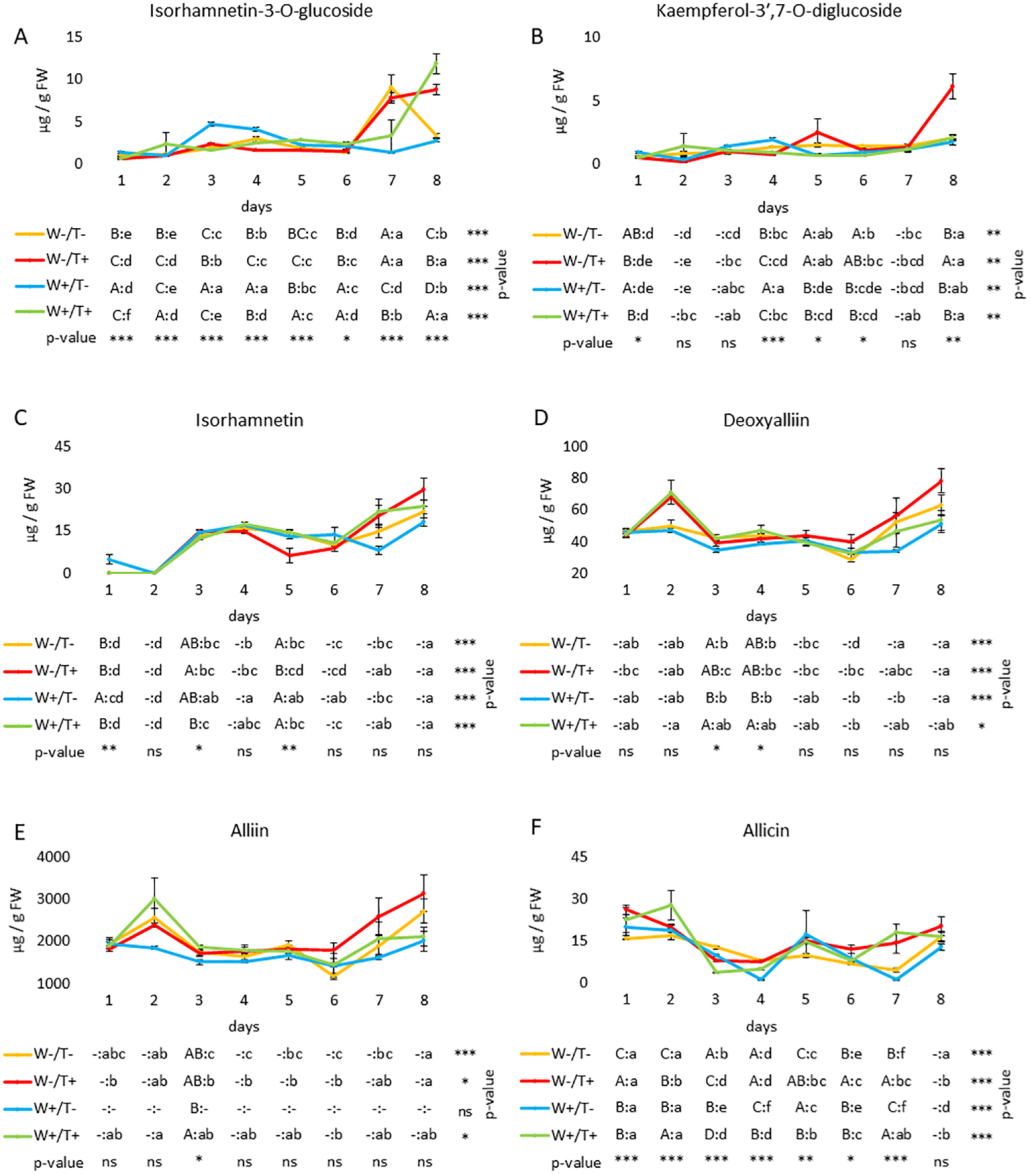
**(A)** Isorhamentin-3-O-glucoside, **(B)** Kaempferol-3′,7-O-diglucoside, **(C)** Isorhamnetin, **(D)** Deoxyalliin, **(E)** Alliin, and **(F)** Allicin content in leaves of garlic plants exposed to water-available and ambient temperature (W+/T-), water-available and high temperature (W+/T+), drought-induced and ambient temperature (W-/T-), and drought-induced and high temperature conditions (W-/T+). Capital letters indicate significant differences between treatments within days in Tukey’s *post-hoc* test, while small letters indicate significant differences between days within treatment groups in Tukey’s *post-hoc* test. ns—not significant; * *p* < 0.05; ** *p* < 0.01; *** *p* < 0.00.

Concurrent with the significantly increased MeJA content towards the end of the experiment, *p*-coumaric acid content was observed to be significantly lower in W−/T−, W+/T+, and W−/T+ plants compared to W+/T− plants, whereas ferulic acid content did not differ among treatments (**Figures 2A, B**). Nonetheless, both *p*-coumaric and ferulic acid contents increased significantly by day 8 relative to the previous days. *p*-coumaric acid content increased only within the watered plants (W+/T- and W+/T+), while ferulic acid content increased within all the treatments, except for day 5 within W-/T- group, and day 4 within the remaining groups (**Figures 2A, B**). Following significantly increased MeJA on days 7 and 8, plants exposed to simultaneous drought and high temperature (W-/T+) on day 7 had significantly higher content of kaempferol-3-O-glucoside, quercetin-3-O-arabinoside, quercetin-3-O-glucoside, quercetin-3,4′-O-diglucoside, and isorhamnetin-3-O-glucoside compared to watered plants at ambient temperature (W+/T-) (**Figures 2C–F**, **3A**). On day 8, in addition to significantly increased kaempferol-3-O-glucoside, quercetin-3-O-arabinoside, quercetin-3-O-glucoside, and isorhamnetin-3-O-glucoside, plants exposed to simultaneous drought and high temperature (W-/T+) also had significantly higher content of kaempferol-3′,7-O-diglucoside compared to watered plants at ambient temperature (W+/T-), while isorhamnetin contents did not differ among treatments (**Figures 3B, C**). Parts of this response were also evident under single-stress conditions. Quercetin-3,4′-O-diglucoside (day 7), isorhamnetin-3-O-glucoside (days 7 and 8), and kaempferol-3-O-glucoside (day 8) content was also significantly higher in W−/T− and W+/T+ plants compared to W+/T− plants (**Figures 2C, F**, **3A**). Moreover, on day 8, quercetin-3-O-glucoside content was significantly higher in W−/T+ compared to W+/T- plants but also compared to W-/T- plants. Similarly, W+/T+ plants had significantly higher quercetin-3-O-glucoside content compared to W+/T− plants, reflecting an effect of temperature, regardless of water availability (**Figure 2E**).

No clear differences between the treatments were observed in deoxyalliin and alliin contents, except for plants exposed to high temperature (W+/T+), which had significantly higher deoxyalliin content on days 3 and 4, and significantly higher alliin content on day 3, compared to watered plants grown at ambient temperature (W+/T-) (**Figures 3D, E**). Plants exposed to either individual (W-/T- and W+/T+) or simultaneous drought and high temperature (W-/T+), had significantly increased allicin content on days 4 and 7 compared to watered plants at ambient temperature (W+/T-) (**Figure 3F**).

### 3.2 Multivariate analysis

**Figure 4:**
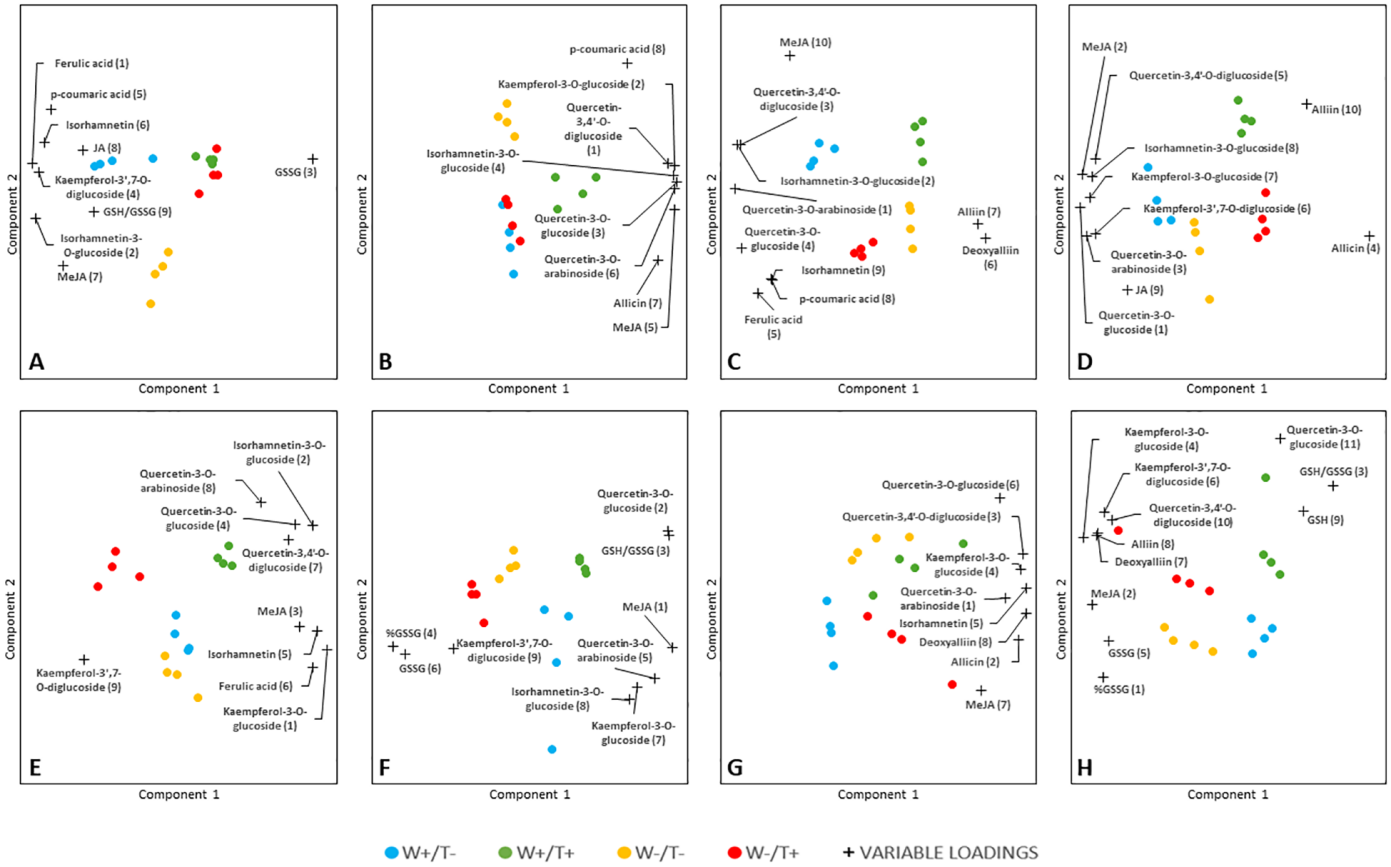
PLS-DA models for the differentiation between the garlic plants exposed to water-available and ambient temperature (W+/T-), water-available and high temperature (W+/T+), drought-induced and ambient temperature (W-/T-), and drought-induced and high temperature conditions (W-/T+) for day **(A)** 1, **(B)** 2, **(C)** 3, **(D)** 4, **(E)** 5, **(F)** 6, **(G)** 7, and **(H)** 8 of exposure. JA, jasmonic acid; MeJA, methyl-jasmonate; GSH, reduced glutathione; GSSG, oxidized glutathione; GSH/GSSG, ratio of reduced glutathione to oxidized glutathione; %GSSG, proportion of oxidized glutathione within the total glutathione pool; W+/T-, water-available and ambient temperature conditions; W+/T+, water-available and high temperature conditions; W-/T-, drought-induced and ambient temperature conditions; W-/T+, drought-induced and high temperature conditions.

The PLS-DA models showed that during the first three days of the experiment no clear effect of water deficit on secondary metabolism was observed, consistent with the lack of significant differences in substrate water content between treatments prior to day 3. Similarly, no clear effect of high temperature on secondary metabolite biosynthesis was detected during this period. Nevertheless, isorhamnetin-3-O-glucoside, quercetin-3,4′-O-diglucoside, and quercetin-3-O-glucoside were identified as key variables contributing to the differentiation of watered plants grown at ambient temperature (W+/T−) from the other treatments (**Figures 4A–C**). The first separation of treatments according to water availability emerged on day 3 along component 2, representing a secondary source of variation. Although higher MeJA content was associated with watered plants (W+/T− and W+/T+) on this day, its contribution to the differentiation between watered and drought-exposed plants was minimal (**Figure 4C**).

By day 4, the discriminatory importance of MeJA increased, and by day 6 MeJA remained one of the most dominant variables in distinguishing watered (W+/T− and W+/T+) from drought-exposed plants (W−/T− and W−/T+) (**Figures 4D–F**). In addition to MeJA, several polyphenolic compounds, including quercetin-3-O-glucoside, isorhamnetin-3-O-glucoside, kaempferol-3-O-glucoside, and quercetin-3-O-arabinoside were also identified by the models as variables contributing to separation between watered (W+/T− and W+/T+) and drought-exposed plants (W−/T− and W−/T+) (**Figures 4D–F**). On day 6, redox-related parameters further contributed towards differentiation between treatments, with higher GSH/GSSG ratio associated with watered plants (W+/T− and W+/T+), while higher %GSSG and GSSG content were characteristic of drought-exposed plants (W−/T− and W−/T+) (**Figure 4F**).

On day 7, the model separated watered plants grown at high temperature (W+/T+) and drought-exposed plants (W−/T− and W−/T+) from watered plants grown at ambient temperature (W+/T−) along component 1 (**Figure 4G**). While higher MeJA content had previously been associated with watered plants, on this day higher MeJA content was linked to plants exposed to simultaneous drought and high temperature (W−/T+). Accordingly, higher quercetin-3,4′-O-diglucoside, quercetin-3-O-arabinoside, kaempferol-3-O-glucoside, isorhamnetin (and allicin) were selected by the model as contributing differentiating factors between plants exposed to high temperature (W+/T+ and W−/T+) and W+/T− plants (**Figure 4G**). By the final day, the PLS-DA model showed clear differentiation of treatments along component 1 according to water availability and along component 2 according to temperature exposure (**Figure 4H**). The model identified %GSSG, MeJA and GSSG contents as key variables in differentiating plants exposed to drought (W−) from watered plants (W+), irrespective of temperature (**Figure 4H**). Under drought conditions, exposure to high temperature further enhanced separation, with kaempferol-3-O-glucoside, kaempferol-3′,7-O-diglucoside, quercetin-3,4′-O-diglucoside, deoxyalliin, and alliin identified as the main discriminating variables (**Figure 4H**).

### 3.3 Exploratory pathway analysis

**Figure 5:**
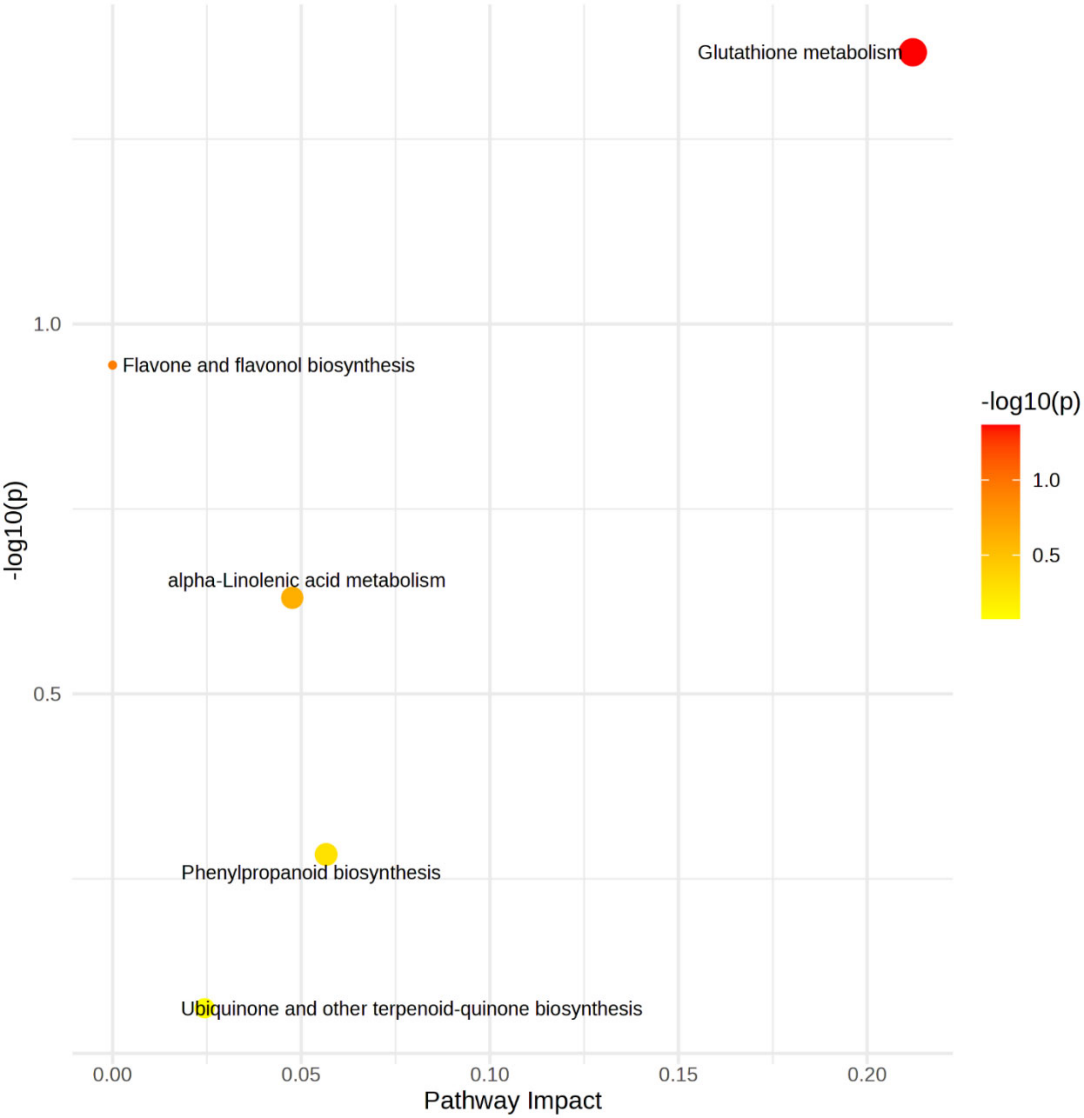
Pathway analysis of garlic redox-, jasmonate-related, organosulfur, and polyphenolic metabolites under abiotic stress. The x-axis represents the pathway impact value computed from pathway topological analysis, and the y-axis is the -log_10_(*p*-value) obtained from pathway enrichment analysis. The pathways that were most significantly changed are characterized by both a high -log_10_(*p*-value) and high impact value.

As an additional exploratory and supportive approach, pathway analysis identified several pathways associated with the targeted metabolite dataset, including *glutathione metabolism, flavone and flavonol biosynthesis, α-linolenic acid metabolism, phenylpropanoid biosynthesis, and ubiquinone and other terpenoid-quinone biosynthesis* (**Figure 5**, **Table 2**). Among these, *glutathione metabolism* emerged as the primary responsive network, exhibiting the highest pathway impact and enrichment, indicating its greater topological relevance within the mapped metabolite network (**Figure 5**, **Table 2**). *Flavone and flavonol biosynthesis* pathway was positioned higher on the y-axis, indicating higher enrichment, but it also showed low pathway impact, while *α-linolenic acid metabolism* and *phenylpropanoid biosynthesis* showed moderate pathway impact with non-significant enrichment values (**Figure 5**, **Table 2**). *Ubiquinone and other terpenoid-quinone biosynthesis* pathway showed the weakest pathway-level indication among the mapped pathways (**Figure 5**, **Table 2**).

**Table 2:** Metabolic pathway analysis of garlic redox-, jasmonate-related, organosulfur, and polyphenolic metabolites under abiotic stress.

Metabolic Pathway	Total (compounds)	Hits (compounds)	Raw *p*-value	-log_10_(*p*)	False Discovery Rate (FDR)	Pathway Impact
Glutathione metabolism	26	2	0.0429	1.367	0.2146	0.212
Flavone and flavonol biosynthesis	10	2	0.1137	0.944	0.2841	0.000
α-Linolenic acid metabolism	26	2	0.2345	0.630	0.3908	0.048
Phenylpropanoid biosynthesis	43	2	0.5214	0.283	0.6517	0.057
Ubiquinone and other terpenoid-quinone biosynthesis	48	1	0.8414	0.075	0.8414	0.024

## 4 Discussion

### 4.1 Univariate analysis of treatment effects

Drought and high temperature stress are one of the most critical abiotic factors limiting crop productivity, as they negatively affect both growth and yield (Hu et al., 2022; Kopecká et al., 2023). One of the prevalent defense responses is regulation of phenolic and flavonoid metabolites, which alleviate oxidative injury by scavenging ROS and maintaining cellular homeostasis (K. Kumar et al., 2023). Numerous studies have shown that both stressors trigger profound metabolic adjustments in different plant species, often involving growth–defense trade-offs and reallocation of resources toward survival and defense (Huot et al., 2014). In our companion paper on garlic plants, we showed that the decrease of substrate water content in combination with high temperature after the fourth experimental day elicited an increase in leaf abscisic acid content and by day 8 a significant increase in leaf proline (Kovačević et al., 2026). The co-occurrence of drought and high temperature, increasingly predicted under climate change scenarios, can result in more severe effects than either stress alone (Zandalinas et al., 2021). This has been demonstrated across multiple plant species, where simultaneous drought and high temperature consistently triggered stronger metabolic perturbations and greater yield reductions, suggesting the presence of conserved but energetically costly defense mechanisms (Zandalinas et al., 2018).

Glutathione is one of the most abundant metabolites in plants, playing crucial roles in ROS detoxification, stress signal transduction, and overall stress adaptation (Foyer and Noctor, 2011). Consequently, maintaining redox homeostasis is a fundamental regulator of both developmental processes and defense mechanisms (Hasanuzzaman et al., 2019). While measurements of GSH and GSSG content can provide information about redox status in plants during abiotic stress, the proportion of GSSG (%GSSG) and the GSH/GSSG ratio serve as more sensitive indicators of oxidative stress, while the latter can also function as a redox signal (Noctor et al., 2012). In the present study, no clear differences in glutathione-related parameters were observed between treatments until day 8 (**Figures 1A–D**), indicating that variations in GSSG content, %GSSG, and the GSH/GSSG ratio during this period cannot be directly attributed to either drought or high temperature. These early fluctuations may reflect transient adjustments in cellular redox homeostasis. Such adjustments are broadly consistent with the initial stress-response processes described by Kranner et al. (2010) (Kranner et al., 2010), in which stress perception and redox-related signaling precede the full activation of protection and repair mechanisms. By the end of experiment, plants exposed to drought (W−/T+ and W−/T−), regardless of temperature, had significantly higher GSSG content, as well as significantly higher %GSSG and significantly lower GSH/GSSG ratio compared to watered plants (W+/T- and W+/T+) (**Figures 1A–D**). Taken together, these results indicate a shift toward a more oxidized cellular environment and redox imbalance, suggesting enhanced ROS-associated signaling and an increased demand for antioxidant defense mechanisms under drought stress (Gill and Tuteja, 2010). Similar results have been reported across different plant species, indicating that drought strongly affects glutathione-mediated antioxidant responses, whereas heat-induced changes in antioxidant and glutathione systems vary with stress intensity, duration, species, and genotype (Hasanuzzaman et al., 2020). The co-occurrence of drought and high temperature can lead to enhanced oxidative stress and stronger activation of redox-buffering systems (Suzuki et al., 2014; Zandalinas et al., 2018).

Phytohormones, particularly jasmonic acid (JA) and its derivatives (MeJA), are associated with the activation of the plant secondary metabolism (Chen et al., 2019; Luo et al., 2023; Savchenko et al., 2019; Wasternack and Strnad, 2019). Different jasmonate derivatives, including JA and its methylated form, MeJA, have distinct biological roles, thus the absence of clear differences in JA content between treatments, contrasted with the pronounced significant differences in MeJA content (**Figures 1E, F**), may reflect functional differentiation within the jasmonate pathway, where MeJA acts as a mobile signaling molecule responsive to stress conditions, according to Wasternack & Feussner (Wasternack and Feussner, 2018). Additionally, experimental studies have provided evidence for MeJA-mediated induction of flavonoid biosynthesis (Premathilake et al., 2020) and for increased accumulation of phenolic compounds in *Allium* species (Wang et al., 2022). This relationship was reflected in the present study, where an increase in MeJA content of plants exposed to simultaneous drought and high temperature (W-/T+) (**Figure 1F**) was observed to coincide with increased contents of several secondary metabolites including kaempferol-3-O-glucoside, kaempferol-3′,7-O-diglucoside, quercetin-3-O-arabinoside (**Figures 2C–E**), quercetin-3,4′-O-diglucoside, isorhamnetin, deoxyalliin, and alliin (**Figures 3A, B, D, E**) compared to watered plants at ambient temperature (W+/T-). The present results revealed treatment- and time-dependent increases in MeJA and several flavonoid and sulfur-related metabolites contents, indicating the need for further studies for the investigation of causal effect of jasmonate-related compounds on early secondary metabolite accumulation in garlic.

Polyphenolic compounds act as key antioxidants in plants by scavenging ROS and protecting cellular components from oxidative damage under abiotic stress. In addition to direct ROS detoxification, they contribute to redox homeostasis and photoprotection, thereby enhancing plant tolerance to environmental stress (Agati et al., 2025). Toward the end of the experimental period, plants exposed to drought at ambient temperature (W−/T−) showed reduced substrate water content compared to watered plants (W+/T−) (**Table 1**), together with significant changes in glutathione-related parameters (**Figures 1B–D**) and increased MeJA content relative to earlier time points (**Figure 1F**). Together, these concurrent changes suggest that W−/T− plants responded to reduced substrate water availability through an interconnected signaling network involving glutathione redox changes and jasmonate-related metabolism (Noctor et al., 2012; Wasternack and Hause, 2013). At the same time, higher contents of several polyphenolic compounds, including kaempferol-3-O-glucoside, quercetin-3-O-glucoside, quercetin-3,4′-O-diglucoside, and isorhamnetin-3-O-glucoside, were observed (**Figures 2C, E, F**, **3A**). Moreover, the contents of kaempferol-3′,7-O-diglucoside and quercetin-3-O-arabinoside of W-/T- plants were comparable to W+/T- plants by the end of experiment, but the content of almost all the investigated polyphenolic compounds increased within W-/T- plants, except for *p*-coumaric acid, quercetin-3-O-arabinoside, and isorhamnetin-3-O-glucoside (**Figures 2A–F**, **3A-C**). The temporal overlap between the redox- and jasmonate-related responses and the increased contents polyphenolic compounds is consistent with previous reports describing links among redox balance, jasmonate-related signaling, and phenylpropanoid metabolism (Falcone Ferreyra et al., 2012).

Polyphenolic compounds are known to accumulate in response to abiotic stresses, but the magnitude, direction, and specificity of these responses can vary depending on the species, genotype, and environmental context, highlighting the complexity of phenylpropanoid pathway regulation under stress conditions (Agati et al., 2012). Thus consequently, individual/total polyphenolic content may exhibit divergent responses under drought, as seen in the present study, as well as in reports for onion (Gedam et al., 2021; Ghodke et al., 2018; Rodrigues et al., 2011), garlic (Bideshki et al., 2013; Habuš Jerčić et al., 2023; Kovačević et al., 2023), shallot (Ghassemi-Golezani et al., 2018), basil (Al-Huqail et al., 2020), *Arabidopsis* (Nakabayashi et al., 2014), and wheat (Laddomada et al., 2021). In the present study, a distinct pattern of polyphenolic accumulation was observed in the selected garlic ecotype (cv. Istarski crveni), while previous studies on this cultivar have reported different trends (Habuš Jerčić et al., 2023; Kovačević et al., 2023). These variations further highlight garlics phenotypic plasticity under differing agroclimatic conditions, as well as differences in experimental approaches, particularly drought simulation methods (PEG versus water deprivation) and stress duration and severity (Poljuha et al., 2021; Wang et al., 2014). Additionally, further complexity arises from the fact that polyphenols comprise a diverse group of interrelated compounds within the phenylpropanoid pathway, where individual metabolites are biosynthetically connected and occur in multiple glycosylated forms with distinct stability, localization, and biological functions (Agati et al., 2012; Falcone Ferreyra et al., 2012), as well as from variations in metabolite extraction, detection, annotation, and quantification methodologies (Alseekh et al., 2021).

According to the results of present study, high temperature was also observed to induce variable effects on content of the investigated polyphenolic compounds, with kaempferol-3-O-glucoside, quercetin-3-O-glucoside, quercetin-3,4′-O-diglucoside, and isorhamnetin-3-O-glucoside being significantly higher in W+/T+ compared to plants at ambient temperature (W+/T-), while no difference in kaempferol-3′,7-O-diglucoside, quercetin-3-O-arabinoside, and isorhamnetin content between the two treatments was observed (**Figures 2C–F**, **3A-C**). Similar results were found in literature *Artemisia sieberi alba* (Alhaithloul, 2019), barley (Karamzadeh et al., 2025), and wheat (Chaouachi et al., 2024; H. Liu et al., 2019) plants. Interestingly, regardless of abiotic stress being drought and/or high temperature, the plants exposed to them (W-/T-, W+/T+, and W-/T+) had significantly lower *p*-coumaric and ferulic acids compared to the watered plants at ambient (W+/T-) towards the end of experiment (**Figures 2A, B**). Although this pattern cannot be interpreted as direct evidence of pathway activation or altered enzyme activity, at the metabolite level, it may indicate a shift in phenylpropanoid metabolism in which hydroxycinnamic acid intermediates did not accumulate as free phenolic acids but were instead more rapidly converted, conjugated, or redirected toward downstream branches such as flavonoid glycoside formation (Świeca, 2015). This interpretation is consistent with the role of *p*-coumaroyl-CoA as a central branch-point intermediate feeding flavonoid biosynthesis, as well as with the broader stress-related role of phenylpropanoid-derived flavonoids in antioxidant protection (Lewis et al., 2024). Therefore, the simultaneous decrease in *p*-coumaric and ferulic acids and increase in flavonoid glycosides could indicate a treatment-associated redistribution within the phenylpropanoid metabolism.

Organosulfur metabolites showed a more variable response than flavonoid glycosides, suggesting that sulfur metabolism was not uniformly affected by short-term unfavorable environmental conditions. Such compound-specific behavior is consistent with the complex biosynthesis and tissue-dependent regulation of garlic organosulfur compounds (Yang et al., 2021). This is biologically plausible because the alliin–allicin system depends on several interconnected processes, including precursor accumulation, enzymatic conversion, and compound stability (Block, 2010). In intact *Allium* tissues, S-alk(en)yl cysteine sulfoxides such as alliin function as sulfur-containing precursor compounds, whereas allicin is formed after tissue disruption through alliinase-mediated conversion of alliin (Borlinghaus et al., 2014). Therefore, changes in alliin, deoxyalliin, and allicin contents do not necessarily reflect a single linear response of the entire sulfur pathway. In the present study, the increase in alliin, deoxyalliin, and allicin within W−/T− plants toward the end of the experiment, together with the absence of clear difference in allicin content between W-/T- and W+/T- plants at ambient temperature (**Figures 3D–F**), may indicate a time-dependent adjustment of sulfur-containing metabolites under drought, rather than a drought-induced upregulation relative to control conditions (Yang et al., 2021). Under high temperature, however, the response was less consistent (**Figures 3D–F**). In W+/T+ plants, alliin and deoxyalliin were comparable to W+/T− plants, while allicin decreased by the end of the experiment compared to the start of the experiment, suggesting that high temperature may have imposed metabolic constraints affecting downstream sulfur-metabolite accumulation more than precursor contents (Hasanuzzaman et al., 2013). Under simultaneous drought and high temperature, organosulfur metabolites in W-/T+ plants showed a divergent pattern. Deoxyalliin and alliin contents were comparable to those of watered plants at ambient temperature (W+/T−), indicating no clear treatment-specific accumulation relative to the control. However, within W−/T+ plants, both precursor compounds increased toward the end of the experiment compared with earlier sampling days, while allicin content decreased during the same period (**Figures 3D–F**). This divergence suggests that concurrent drought and high temperature did not uniformly enhance sulfur-metabolite accumulation, but rather affected different components of the alliin–allicin system in a compound-specific and time-dependent manner (Borlinghaus et al., 2014; Yang et al., 2021). Since allicin accumulation depends not only on precursor availability but also on conversion efficiency and compound stability, the opposite temporal patterns of alliin/deoxyalliin and allicin may reflect metabolic constraints on downstream sulfur-metabolite accumulation under co-occurring drought and high temperature stress (Block, 2010). These findings highlight the importance of considering interacting stress factors in *Allium* cultivation, as temperature-mediated effects on polyphenolic and sulfur metabolism may be as critical as water availability in determining crop quality. Further studies are needed to elucidate causal relationships in the long-term adaptation of garlic secondary metabolism under single and co-occurring drought and high temperature stresses.

### 4.2 Multivariate approach to early metabolic response pattern discovery

Optimal conditions, such as sufficient water supply and adequate ambient temperature, support stable photosynthetic activity and sufficient carbon flux, which in turn may provide favorable conditions for the accumulation of phenylpropanoid-derived compounds (Ninkuu et al., 2025). Although during the first three days of the experiment the PLS-DA models did not indicate a clear separation pattern attributable to either water deficit or high temperature treatment, higher contents of MeJA, isorhamnetin-3-O-glucoside, quercetin-3,4′-O-diglucoside, and quercetin-3-O-glucoside contributed to the differentiation of watered plants grown at ambient temperature (W+/T−) from the other treatments (**Figures 4A–C**).

By day 4, MeJA content became a more prominent variable contributing to the separation between watered plants (W+/T− and W+/T+) and drought-exposed plants (W−/T− and W−/T+), increasing from the 10^th^ to the 2^nd^ most influential variable in the model, a trend that remained visible until day 7 (**Figures 4D–F**). During this period, higher GSH/GSSG ratio, and higher contents of quercetin-3-O-glucoside, isorhamnetin-3-O-glucoside, kaempferol-3-O-glucoside, and quercetin-3-O-arabinoside, together with lower %GSSG and GSSG content, were also associated with the separation of watered (W+/T− and W+/T+) and drought-exposed (W−/T− and W−/T+) plants (**Figures 4D–F**). Trends observed during this period of the experiment indicate that treatment differentiation was accompanied by changes in redox-related parameters and flavonoid glycosides.

On day 7, the model separated watered plants grown at high temperature (W+/T+) and drought-exposed plants (W−/T− and W−/T+) from watered plants grown at ambient temperature (W+/T−) along component 1 (**Figure 4G**). A similar separation trend was observed in the primary metabolism described in the companion paper as well (Kovačević et al., 2026), suggesting that, by day 7, stress-related differences were more clearly reflected across multiple metabolite classes. While higher MeJA content was previously associated with watered plants, from day 7 onward it was more closely associated with plants exposed to simultaneous drought and high temperature (W−/T+). MeJA is widely recognized as a stress-responsive signaling molecule involved in plant defense and secondary-metabolite regulation under unfavorable environmental conditions (Davies, 2010; Kazan, 2015; Wasternack and Feussner, 2018; Wasternack and Hause, 2013). According to the day 7 PLS-DA model, higher contents of quercetin-3,4′-O-diglucoside, quercetin-3-O-arabinoside, kaempferol-3-O-glucoside, isorhamnetin, and allicin were associated with W+/T+ and W−/T+ plants and contributed to their separation from W+/T− plants (**Figure 4G**). This indicates that these compounds were important contributors to the treatment-specific metabolic profiles observed under high temperature, particularly when combined with drought. This shift in metabolite accumulation trend is consistent with contemporary growth–defense trade-off frameworks, according to which stress conditions may alter resource allocation away from growth and toward secondary metabolism and defense-related compounds (Huot et al., 2014).

Rapid and intense ROS increase can be associated with heat stress (Fortunato et al., 2023; Hasanuzzaman et al., 2020), whereas responses to drought or combined drought and high temperature often develop progressively as metabolic reprogramming unfolds over time (Krasensky and Jonak, 2012). Accordingly, higher contents of polyphenolic compounds were initially associated with W+/T- from day 1 to day 6, followed by W+/T+ plants on days 2, 5, 6, and 7. By days 7 and 8, higher content of individual polyphenolic compounds became more evident in W-/T+ plants, while W-/T- plants showed this trend on day 8. Observed temporal shift suggests that the contribution of secondary metabolites to treatment differentiation was both time- and treatment-dependent. Additionally, in drought-exposed plants (W-/T- and W-/T+), early metabolic responses may have been more closely linked to osmotic adjustment before greater accumulation of secondary metabolites such as phenylpropanoid-derived compounds became evident later in the experiment (M. Kumar et al., 2021).

### 4.3 Exploratory pathway-level mapping of targeted metabolites

Although none of the mapped pathways reached statistical significance based on raw or FDR-adjusted *p*-values, pathway-level mapping was used to provide biological context for the targeted metabolite results. The high pathway impact of *glutathione metabolism* is consistent with the established role of glutathione in antioxidant defense, detoxification, redox homeostasis, and stress-associated signaling in plants (Noctor et al., 2012). Similarly, the mapping of *flavone and flavonol biosynthesis* and *phenylpropanoid biosynthesis* corroborates the observed accumulation of several flavonoid glycosides, and is in line with previous reports describing phenylpropanoid-derived metabolites as important components of abiotic stress protection (Sharma et al., 2019). The appearance of *α-linolenic acid metabolism* is also consistent with the involvement of jasmonate-related metabolism, given its connection to jasmonate biosynthesis and stress signaling (Ali and Baek, 2020). The mapping of *ubiquinone and other terpenoid-quinone biosynthesis* pathway, although weak and not statistically significant, is biologically consistent with the roles of ubiquinone-related metabolites in electron transport and antioxidant protection during plant stress (M. Liu and Lu, 2016). Because targeted LC-MS/MS measures a selected metabolite subset, pathway coverage is incomplete. Therefore, pathways may show meaningful biological alignment with measured results even without significant FDR values, but they should be interpreted as supportive pathway mapping rather than statistically significant pathway enrichment (Wieder et al., 2021).

Overall, the findings suggest associations at the metabolite-level among glutathione redox status, jasmonate-related metabolism, phenylpropanoid-derived compounds, and organosulfur metabolites. Furthermore, the results highlight glutathione redox parameters and MeJA as candidate metabolite-level indicators of early garlic responses to reduced water availability and high temperature. However, their use in stress monitoring requires further validation across field conditions, developmental stages, and garlic genotypes.

## Data Availability

The raw data supporting the conclusions of this article will be made available by the authors, without undue reservation.
